# Ameliorative Effect of Pomegranate Peel Extract (PPE) on Hepatotoxicity Prompted by Iron Oxide Nanoparticles (Fe_2_O_3_-NPs) in Mice

**DOI:** 10.3390/nano12173074

**Published:** 2022-09-04

**Authors:** Yasmin M. Abd El-Aziz, Basma M. Hendam, Fawziah A. Al-Salmi, Safa H. Qahl, Eman H. Althubaiti, Fahmy G. Elsaid, Ali A. Shati, Nasser M. Hosny, Eman Fayad, Ali H. Abu Almaaty

**Affiliations:** 1 Department of Zoology, Faculty of Science, Port Said University, Port Said 42526, Egypt; 2 Department of Husbandry & Development of Animal Wealth, Faculty of Veterinary Medicine, Mansoura University, Gomhoria St., Mansoura 35516, Egypt; 3 Department of Biology, Faculty of Sciences, Taif University, Taif 21944, Saudi Arabia; 4 Department of Biology, College of Science, University of Jeddah, Jeddah 21589, Saudi Arabia; 5 Department of Biotechnology, Faculty of Sciences, Taif University, Taif 21944, Saudi Arabia; 6 Biology Department, Science College, King Khalid University, Abha 61421, Saudi Arabia; 7 Zoology Department, Faculty of Science, Mansoura University, Mansoura 35516, Egypt; 8 Department of Chemistry, Faculty of Science, Port Said University, Port Said 42526, Egypt

**Keywords:** iron oxide nanoparticles (Fe_2_O_3_-NPs), pomegranate peel extract (PPE), light microscopy, histochemistry, chromosomal aberrations, gene expression, antioxidant biomarkers

## Abstract

An evaluation of the ameliorative effect of pomegranate peel extract (PPE) in counteracting the toxicity of iron oxide nanoparticles (Fe_2_O_3_-NPs) that cause hepatic tissue damage is focused on herein. Forty male albino mice were haphazardly grouped into four groups as follows: the first control group was orally gavage daily with physiological saline; the second group received 100 mg/kg of PPE by the oral route day after day; the third group received 30 mg/kg Fe_2_O_3_-NPs orally; and the fourth group received both PPE and Fe_2_O_3_-NPs by the oral route, the same as the second and third sets. Later, after the completion of the experiment, we collected the liver, blood, and bone marrow of bone specimens that were obtained for further laboratory tests. For instance, exposure to Fe_2_O_3_-NPs significantly altered serum antioxidant biomarkers by decreasing the levels of total antioxidant capacity (TAC), catalase (CAT), and glutathione s-transferase (GST). Additionally, it caused changes in the morphology of hepatocytes, hepatic sinusoids, and inflammatory Kupffer cells. Furthermore, they significantly elevated the number of chromosomal aberrations including gaps, breaks, deletions, fragments, polyploidies, and ring chromosomes. Moreover, they caused a significant overexpression of TIMP-1, TNF-α, and BAX mRNA levels. Finally, the use of PPE alleviates the toxicity of Fe_2_O_3_-NPs that were induced in the hepatic tissues of mice. It is concluded that PPE extract has mitigative roles against the damage induced by Fe_2_O_3_-NPs, as it serves as an antioxidant and hepatoprotective agent. The use of PPE as a modulator of Fe_2_O_3_-NPs’ hepatotoxicity could be considered as a pioneering method in the use of phytochemicals against the toxicity of nanoparticles.

## 1. Introduction

There are increased daily uses of nanotechnology in many aspects of our lives, including agriculture, industry, medicine, and public health [1]. Additionally, the production of these nanoparticles drastically modifies their chemical, physical, and biological characteristics [2]. Nanoparticles are known to have a few negative consequences in addition to their many positive ones. Although they are used in medicine, there is a worry that they could be harmful to people’s health [3]. The liver, spleen, and lungs are the primary organs where nanoparticles cluster, depending on the distribution pathway [4]. For the most part, hazardous substances such as iron nanoparticle toxicity are assumed to be detoxified by the liver [5].

Due to iron oxide nanoparticles’ (Fe_2_O_3_-NPs) magnetic turf on the exterior, tissues were placed and heated in a discontinuous attractive field because of the high bioavailability of nanoparticles [6]. Due to their nano-size and excellent surface-area-to-volume ratios, Fe_2_O_3_-NPs have recently attracted the attention of researchers [7]. Because of their ability to penetrate cells and tissues, they are used successfully in the delivery of genes and medications. Their characteristics have the capacity to be harmful [8]. Additionally, it has been demonstrated that Fe_2_O_3_-NPs are toxic to hepatic tissues and harm the liver [9]. The liver is the primary organ for identifying the adverse effects of in vivo exposure to Fe_2_O_3_-NPs and has the highest concentration of concentrated nanoparticles [5].

Additionally, chronic inflammatory liver injury [10], which is characterized by an accumulation of the extracellular matrix (ECM) that the tissue is remodeling, results in pathological processes such as liver fibrosis and excessive apoptotic syndromes. The tissue inhibitors of matrix metalloproteinases (TIMPs) and MMPs themselves are among the connective tissue breakdown pathways that are dysregulated [11]. TIMP-1 has been identified as a marker for liver fibrosis as a result, and its presence implies an increase in liver damage [12]. Additionally, it impairs liver regeneration and encourages liver fibrosis [13].

A pleiotropic inflammatory cytokine called tumor necrosis factor-alpha (TNF-α) influences apoptosis, immune responses, and other cellular and biological processes [14]. Monocytes and macrophages are a couple of immune cells that are involved [15]. Increased TNF production causes an uptick in inflammatory and oxidative responses [16]. Numerous hepatic illnesses’ vulnerability, as well as their origin and development, have been linked to polymorphisms in the TNF-α gene [17]. It also accelerates the demise of hepatocytes and their differentiation [18] and augments the progression of liver fibrosis [19].

Since its homodimerization and the consequent creation of holes in the mitochondrial external membrane are necessary for apoptotic cell death, BAX is also recognized as a crucial pro-apoptotic protein. Along with Bcl-2, it can also form heterodimers, which help to block its main anti-apoptotic action [20]. Pro- and anti-apoptosis genes work together to alter cell strength during the apoptosis process [21,22]. People with liver disease had significantly higher levels of the BAX gene, showing that hepatic steatosis and fibrosis affect the expression of pro- and anti-apoptotic proteins [17].

Due to their many health benefits, pomegranates (*Punica granatum*) have long been grown and enjoyed as delicious fresh fruit. Both the fruit juice and peel from pomegranates are rich in antioxidants [23]. It is a tree in the *Lythraceae* family that is deciduous [24]. Furthermore, due to the antioxidant and free-radical scavenging properties of their bioactive components, the polyphenols in the pomegranate’s primary components lessen lipid peroxidation [25]. The most significant class of bioactive chemicals found in plants are thought to be polyphenols. They possess antioxidant, anti-inflammatory, and anti-allergic activities, and they have a particular structural characteristic that consists of one or more aromatic phenyl rings connected to hydroxyl groups [26]. This study sought to ascertain the beneficial role of pomegranate peel extract (PPE) in mitigating the negative impacts of Fe_2_O_3_-NPs using a variety of biochemical, histological, and genetic traits.

This study aimed to evaluate the ameliorative effects of pomegranate peel extract (PPE) against the hepatotoxicity of Fe_2_O_3_-NPs through the assessment of some antioxidant biomarkers, hepatic morphologic changes, chromosomal analysis, and mRNA expression patterns of TIMP-1, TNF-α, and BAX genes.

## 2. Materials and Methods

### 2.1. Ethical Statement

The animal study was approved and reviewed by the Institutional Animal Care and Animal Ethics Committee, Faculty of Science, Port Said University, Port Said, Egypt. All precautions and guidelines were followed to minimize animal suffering and ensure animal welfare during the experiment.

### 2.2. Preparation of Fe_2_O_3_ Nanoparticles (Fe_2_O_3_-NPs)

We mixed the FeCl_3_·6H_2_O solution (2.7 g dissolved in distilled H_2_O) with 1.3 g of anthranilic acid in distilled water with continuous stirring. Then, we added 1.0 M K_2_Cr_2_O_7_ (18 mL) to the reaction mixture, stirring for about one hour. We kept the mixture overnight, then added NH_4_OH (1:1) until precipitation. In addition, we filtered the precipitate and washed it with distilled water and alcohol; then, we dried it at 70 °C for 2 h. Then, it burnt nearly 2 gm of dried precipitate at 800 °C in the air [27].

#### XRD and TEM Characterization of Fe_2_O_3_-NPs

XRD was carried out on a Philips X’PERT-PRO diffractometer, (λ = 1.5406 Ǻ for Cu K α radiation). TEM images were recorded by a JEOL JEM-1200EXII microscope (Tokyo, Japan).

### 2.3. Procedures of Pomegranate Peel Extract Preparation (PPE)

Pomegranate fruits were obtained from the fruit supermarket in Port Said city, Port Said Province, Egypt. The fruits were washed carefully and peeled within a day. The pomegranate peel extract (PPE) was prepared through the following procedures: briefly, the fresh pomegranate peels were separated manually and dried naturally for ten days; then, they were put into a freezer dryer and crushed using grindery (Retsch, Haan, Germany). Then, about 200 gm of pomegranate peel was produced after grinding, and then about 50 gm of pomegranate peel powder was mixed with 500 mL of methyl alcohol for 24 h at 22 °C. In addition, the mixed solution of methanolic pomegranate peel powder was filtered twice. Finally, this mixture was evaporated at 50 °C using rotary evaporation (Rotavapor, Stuart, England) [28]. The resulting raw PPE was then red-balanced to 500 gm and kept in a deep freezer at 5 °C until further use in our experimental study.

### 2.4. Experimental Animals

**Figure 1:**
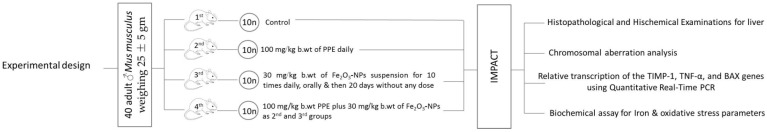
Schematic figure for explaining the experimental design.

Forty adult male albino mice (*Mus musculus*) weighing 25 ± 5 gm were bought from the laboratory animals’ house (Faculty of Science, Port Said University, Port Said, Egypt). The animals were permitted to be adapted for about 14 days below the hygienic laboratory standards at an average temperature rate of 25 ± 1 °C. The other environmental factors of water and food were available to them ad libitum, as they were retained in cages of plastic under an organized periodical cycle (12:12 dark: light), an appropriate humidity rate (51 ± 4%), and good ventilation. Then, the mice were allocated equally to four experimental sets, with ten mice in each one of them, as illustrated in Table 1 and Figure 1. In the current experimental study, we used 100 mg of PPE/kg b. wt. of mice [28]. This extract was gavage orally to the experimental mice for about four weeks. In addition, 30 mg of Fe_2_O_3_-NPs/kg b. wt. of mice was suspended in 100 µL of saline solution (0.98% NaCl) and then separated into ten doses that were administered daily during the first 10 days by a stomach feeding tube, and the remaining 20 days were without receiving Fe_2_O_3_-NPs [29]. The experimental period is 30 days. After 24 h from the last doses, the merciful slaughter was performed.

### 2.5. Histopathological Examinations

#### 2.5.1. Examination of Liver Tissues via a Light Microscope

The liver tissues were quickly removed from each experimental mouse, as they were used for bright routine microscopic examinations, and the liver tissues were fixed with about 10% formalin solution, dehydrated, and cleared. The rest of the hepatic tissue was processed for cutting into thin slices without causing damage to any liver tissue. We used the microtome to embed the hepatic specimens in blocking paraffin wax and cut the slices at a thickness of 5 mm (Leica, Wetzlar, Germany). In addition, the Hx&E stain was utilized to detect the normal and diseased components of the hepatic tissues in liver slices. The liver sections were viewed under a brilliant microscope (Leica, Wetzlar, Germany) after staining [30].

#### 2.5.2. Detection of Liver Tissues Using the Transmission Electron Microscope

The liver tissues were quickly removed and examined using an electron microscope according to the previous study [31]. After that, the hepatic tissues were inspected by a transmission electron microscope (JEOL JEM-2100, Tokyo, Japan) at an accelerating voltage of 80 kV at the Electron Microscopy Unit at the Faculty of Agriculture, Mansoura University, Mansoura, Egypt. 

### 2.6. Histochemical Examinations

Connective tissues, collagenous fiber, reticle, and amyloid were detected in the hepatic cords of liver slices, using the Mallory trichrome reaction (MTS) as specific histochemical stains. After staining liver slides with this specific dye, they were examined using a standard brilliant microscope (Leica, Wetzlar, Germany) [30].

### 2.7. Apoptotic Evaluations

For apoptotic cellular evaluation, we used the TUNEL positive technique, which is present in the hepatic cords of liver slices. Following the staining of liver slides with this specific dye, the slides were examined under a light microscope (Leica, Wetzlar, Germany) [30].

### 2.8. Morphometric Quantifications

The computer system for morphometric evaluation analysis is part of the microsystems for image solutions (Leica, Cambridge, UK). To determine the percentage of collagen fibers in the liver sections, we utilized Mallory trichrome stain on all the collagen fibers. Finally, by collecting random readings from ten liver sections for all experimental mice, the examined results were reported as averages of percentages (fibrous area percent) and SE (standard error) [32].

### 2.9. Chromosomal Aberration Analysis

Rapidly, the femurs and humeral bones of two mice were removed. These bones were cleaned out of the muscles or cartilaginous processes. According to the procedural techniques of Singh and Sankhla [33], the bone marrow was removed from each mouse’s bones. Then, we prepared 5% Giemsa stain, which was used to stain the slides for about 10–15 min. Then, these slides were left to dry well before the examination. For analysis, nearly 100 well-spread slides per mouse were numbered and distinguished for analysis. The determination of any chromosomal disorders occurs by the examination of 500 metaphase chromosomes under light microscopy (Leica, Wetzlar, Germany).

### 2.10. Relative Transcription of the TIMP-1, TNF-α, and BAX Genes Using Quantitative Real-Time PCR

#### 2.10.1. Extraction of RNA and Reverse Transcription

The RNeasy Minute Kit (iNtRON Biotechnology, Seongnam, Korea) was used to extract the total RNA from the liver tissues of the experimental mice, and the quantity and integrity of the recovered RNA were assessed using the NanoDrop^®^ ND-1000 Spectrophotometer. The Sensi FASTTM cDNA synthesis kit (Bioline, London, UK) was used to reverse transcribe RNA into cDNA from each sample according to the manufacturer’s instructions. The cDNA samples were then stored at −20 °C until they were needed.

#### 2.10.2. Quantitative Real-Time PCR

The TIMP-1, TNF-α, and BAX mRNA levels were quantified using the Stratagene MX3005P real-time PCR thermocycler (Agilent Technologies, Santa Clara CA, USA) and the Sensi FastSYBRLo-Roxkit (Bioline, London, UK). In this regard, specific forward and reverse primers and their appropriate NCBI Gen Bank accession numbers for each gene are presented in Table 2. Real-time PCR amplifications were completed in 25 μL of the total reaction mixtures that included 2 μL of the reversed cDNA, 1 μL of each specific primer for each gene (forward and reverse), 12.5 μL of Lo-RoxSYBR, and 8.5 μL of RNase-free H_2_O. The thermal cycling conditions were: denaturation at 95 °C for about 10 min, 40 cycles at a temperature of 95 °C for 15 s and 60 °C for 60 s, and, finally, elongation at 72 °C for 30 s. The melting curves were generated after the end of the qRT-PCR cycles to identify the precise amplification of each target gene product of interest. The relative expression of the gene in each sample was normalized in comparison to the *GAPDH* gene and calculated according to the 2^−ΔΔCt^ method [34]. 

### 2.11. Biochemical Assay

After fasting the mice for about 12 h at the end of the study period (one month), blood samples were collected from retro-orbital venous plexus punctures of the eyes of ten mice from each group under ether anesthesia. These blood samples were left to clot at room temperature for about 15 min. Then, they were centrifuged at 4000 rpm for 15 min. The uppermost supernatant layers were pipetted into another clean Eppendorf tube and stored at −20 °C until further use in the measurement of biochemical parameters with the Perkin Elmer Lambda EZ201 spectrophotometer, Berlin, Germany. After that, all the experimental mice were euthanized by cervical dislocation.

Iron ion metal parameter (Fe), as well as total antioxidant capacity (TAC), catalase (CAT), and glutathione-s-transferase (GST), are antioxidant biomarkers that can be measured. We used the commercial kits that were applied by Bio-Diagnostic Company (Giza, Egypt) according to the TAC, CAT, and GST kits’ pamphlets of the manufacturers’ procedures.

### 2.12. Statistical Analysis

Statistical analyses of the resulting data were performed via one-way Analysis of Variance (ANOVA) through the statistical program SPSS and the Graph Pad Prism Software (“One-way ANOVA followed by Dunnett’s multiple comparisons test was performed using GraphPad Prism version 8.0.0 for Windows, GraphPad Software, San Diego, CA, USA, www.graphpad.com”.) to evaluate the significant differences between all the experimental groups and compare them with the control group. Tukey’s multiple comparison post hoc test was used to make a comparison between the means. The statistical significance levels were accepted at *p* < 0.05, and all the analyzed data were expressed as means ± SE (standard error) [32].

## 3. Results

### 3.1. Characterization of α-Fe_2_O_3_ Nanoparticles (Fe_2_O_3_-NPs)

**Figure 2:**
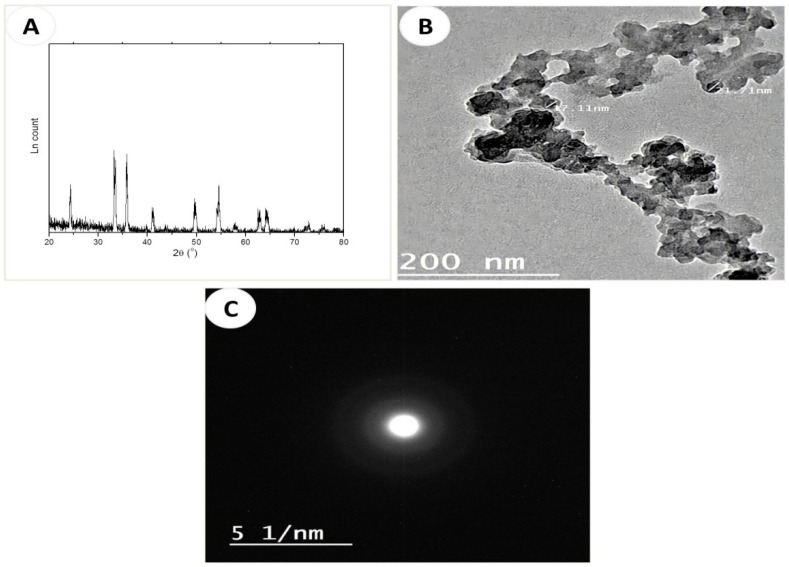
Characterization of Fe_2_O_3_-NPs including: (**A**) XRD, (**B**) TEM image, and (**C**) SAED of Fe_2_O_3_-NPs.

The XRD pattern of the Fe_2_O_3_-NPs (Figure 2A SI) indicated diffraction peaks at 24.0, 33.2, 35.7, 41.0, 49.7, 54.5, 58.0, 62.8, and 64.0°. These peaks corresponded to the planes: 012, 104, 110, 113, 024, 116, 018, 214, and 300, respectively. These findings confirmed that the material was α-Fe_2_O_3_, which crystallized in a hexagonal structure with the lattice constants a = 4.9876, b = 4.9876, and c =13.7489 Å, α = β = 90°, γ = 120° (JCPDS Card No. 01-079-007). The crystallite size of the prepared Fe_2_O_3_-NPs was determined from the major peak at 32.2° by applying the Scherrer formula (D = 0.89 λ/β cosθ, where λ = 1.5406 Ǻ, β is the width at half maximum, and θ is the peak position) [28] and was found to be 26 nm.

The TEM image of the Fe_2_O_3_-NPs (Figure 2B) revealed the formation of irregular spherical particles that resulted from the aggregated nanoparticles with particle sizes in the range of 10–27 nm, which was close to the calculated form from XRD. SAED (Figure 2C) indicated three rings that corresponded to the planes 111, 220, and 311, respectively, thus confirming the formation of Fe_2_O_3_-NPs.

### 3.2. Histopathological Investigations

#### 3.2.1. Studying the H&E Sections by Light Microscopy (LM)

**Figure 3:**
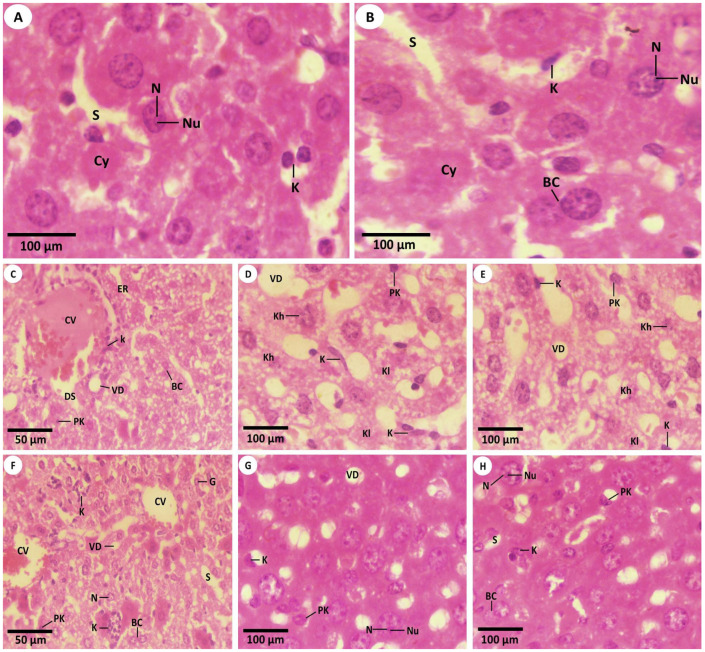
Light histopathological investigation in the liver tissues of control mice (first group), PPE-treated mice (second group), mice exposed to Fe_2_O_3_-NPs (third group), and Fe_2_O_3_-NPs plus PPE-treated mice (fourth group) (**A**) Mouse liver section from the first group displays the healthy tissue that shows the normal structure of liver cells with a normal nucleus (N) and nucleolus (Nu), clear cytoplasm (Cy), Kupffer immune cells (K), and normal blood hepato-sinusoids (S) (Hx.E., 1000×). (**B**) Mouse liver from the second group that displays the normal structure of the clear cytoplasm (Cy) of hepatocytes, Kupffer cells (K), binucleated cells (BC), intact hepatocytes with normal nuclei (N) and a normal nucleolus (Nu), and normal blood hepato-sinusoids (S) gaps (Hx.E., 1000×). (**C**) Mouse liver section from the third group that revealed coagulated hepatocytes with necrosis that appeared densely eosinophilic with the pyknosis stage (PK), endothelial erosion (ER) located in the boundary of the central vein, vacuolar degeneration (VD), the dilation of the blood liver sinusoids (DS), the distortion of the central vein (CV), Kupffer cells (K), and binucleated (BC) (Hx. E., 400×). (**D**) Magnified field of the mouse hepatic slide that appeared in the third group that shows obvious vacuolar degeneration (VD), pyknotic cells in the necrosis stage (Pk), karyorrhexis (Kh), karyolysis (Kl) hepatocytes, and many Kupffer immune swollen cells (K) (Hx. E., 1000×). (**E**) Another magnified field from the mouse hepatic section in the third group that displays the coagulated necrosis of hepatocytes, densely esinophilic nuclei with pyknosis (PK), karyorrhexis (Kh), karyolysis (Kl) of hepatocytes, many Kupffer immune cells (K), and vacuolar degeneration (VD) (Hx. E., 1000×). (**F**) Mouse liver section from the fourth group that displays the restoration of normal hepatic cords with mildly degenerated hepatocytes around the central vein (CV), a vacuolated cytoplasm, the widening of hepatic blood sinusoids (S), intact single nuclei (N) or binuclei (BC), reduced pyknotic cells (Pk), vacuolar degeneration (VD), a hepatic giant cell (G), and Kupffer immune cells (K) (H. E., 400×). (**G**) Magnified field from the mouse hepatic section of the fourth group that shows a recovery in some hepatic strands, mild restoration hepatocytes with nuclei (N) and the nucleolus (Nu), pyknotic cells (Pk) that are decreased significantly, mild vacuolar degeneration (VD), and Kupffer cells (K) (Hx. E., 1000×). (**H**) Another magnified field from the mouse hepatic section that was obtained from the fourth group that displays a restoration of hepatic cords with the mild recovery of hepatocytes with nuclei (N) and the nucleolus (Nu), the reduction of pyknotic hepatocytes (Pk), the mild widening of hepatic blood sinusoids (S), Kupffer cells (K), and binucleated (BC) (Hx. E., 1000×).

The intact texture of the mice′s livers from the control (first) group was noticed in Figure 3A. Normal liver cells were presented to be with a single rounded or oval nucleus or binucleated nuclei. Additionally, there were no distinguishable histological alternations in the hepatocytes of the hepatic tissues in the mice that received the PPE (second group) as compared to the livers from the first group (Figure 3B). Meanwhile, the liver tissues of Fe_2_O_3_-NPs (third group) revealed the coagulative necrosis of hepatic cells, as presented by dense pyknotic nuclei and dilated blood vessels of hepatic sinusoids with erosions in the endothelial lining that surrounded the central hepatic vein. Numerous Kupffer cells were revealed. Additionally, obvious vacuolar degeneration and necrosis stages appeared as pyknosis, karyorrhexis, and karyolysis of hepatocytes. In addition, it was noticed that there were numerous swollen immune Kupffer cells in the Fe_2_O_3_-NPs intoxicated group (Figure 3C–E). On the other hand, it was displayed that the liver cells of the mice that received both PPE and Fe_2_O_3_-NPs (fourth group) showed a degree of improvement in the architecture as compared with the mouse liver cells of the third group (Figure 3F). The hepatocytes displayed an intact structure with a normal hepatic central vein, along with some vacuolization of the narrow blood hepatic sinusoids (Figure 3G,H). The mice′s livers appeared with particular hepatic strands, with more restoration viewed in liver tissues. Intact nuclei (either single or binuclei), some pyknosis of the hepatic cells, and a certain swelling of immune Kupffer cells were also noticed (Figure 3F–H).

#### 3.2.2. Investigation of the Liver Ultrastructure by a Transmission Microscope

**Figure 4:**
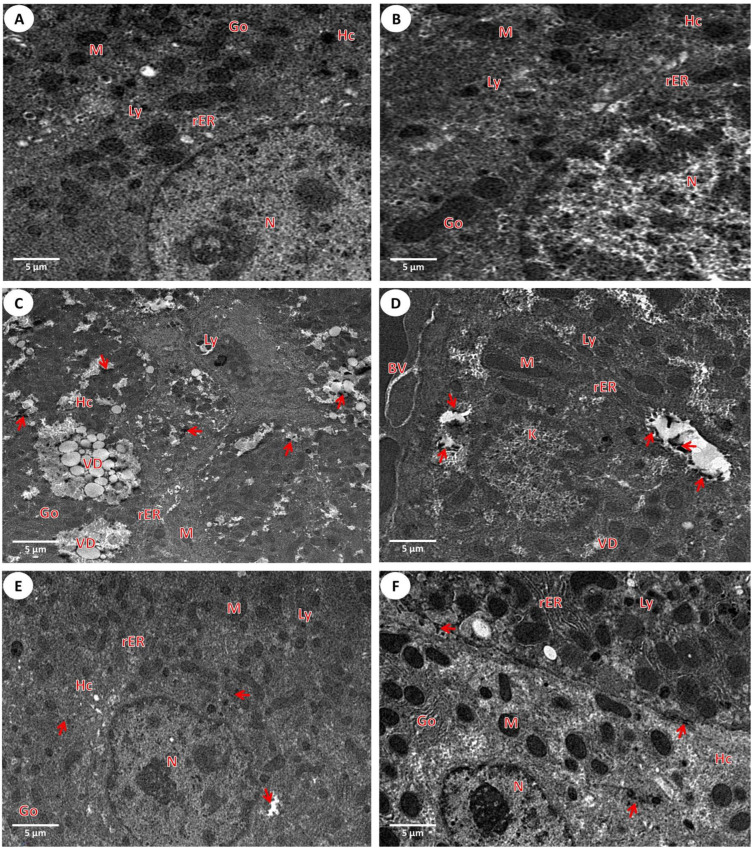
Electron examination of liver slices for experimental groups: (**A**) Hepatic section from the first group (control), (**B**) Hepatic section from the second group (PPE), (**C**,**D**) Hepatic section from the third group (Fe_2_O_3_-NPs), and (**E**,**F**) Hepatic section from the fourth group (PPE plus Fe_2_O_3_-NPs). The ultrastructures show the hepatic cells (Hc), the nucleus (N) of hepatocytes, the Kupffer cell (K), the mitochondria (M), the Golgi apparatus (Go), the rough endoplasmic reticulum (rER), the lysosome (Ly), the vacuolar hepatic degeneration (VD), and the iron precipitate (arrows) in the slices (15,000×).

Ultrathin liver slices from the first group (control) showed normal hepatic cells with normal nuclei that are represented in Figure 4A. Meanwhile, as show in Figure 4B, the slices from the second group (PPE) revealed the normal liver structure that resembled the ultrathin liver of the first group. On the other hand, the hepatic slices from the third group (Fe_2_O_3_-NPs) represented the iron precipitate inside the hepatic tissues, the vacuolar hepatic degeneration form, some hepatic cells in the necrosis stage, the damage of the mitochondria, and lysosomes that increased in size and were numerous inside cells (Figure 4C,D). Finally, the transmission ultrastructure of the liver tissues from the fourth group (PPE and Fe_2_O_3_-NPs) explained the improvement of the ultrathin liver with some ameliorate structures of the liver as hepatic cells, the improvement of the hepatocytes, the mitochondria, the number of lysosomes, and the Kupffer cells (Figure 4E,F). 

#### 3.2.3. Histochemical Investigations and Morphometric Quantification

**Figure 5:**
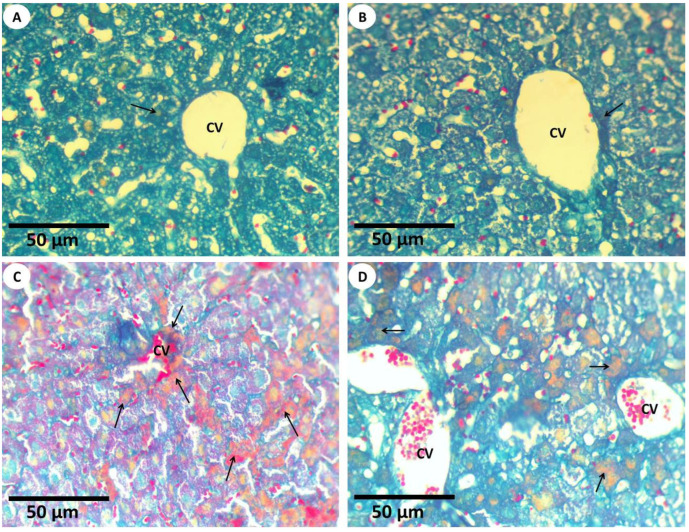
Light explanations microscopy for histochemistry fiber studies (**A**) Mouse liver section from the first group displaying the healthy tissues and the normal distribution of collagen and reticular fibers (arrow) in the intact liver strands around the central vein (CV) (MTS, 400×). (**B**) Mouse liver section from the second group showing the normal distribution of collagenous fibers (arrow) in the hepatic cord that was found around the central vein (CV) using Mallory trichrome stain (MTS, 400×). (**C**) Mouse liver section from the third group that displays amyloidosis and collagenous fibers (arrows) that were aggregated in the liver tissues after the mice were exposed to Fe_2_O_3_-NPs; the central vein (CV) was also engorged with blood (MTS, 400×). (**D**) Mouse liver section from the fourth group showing an improvement in the collagen fibers (arrows) within the hepatocyte, which were located around the hepatic central veins (CV) (MTS, 400×); this shows the usefulness of using PPE in the improvement of toxicity, which was induced in the morphology of the liver tissues of mice exposed to Fe_2_O_3_-NPs.

**Figure 6:**
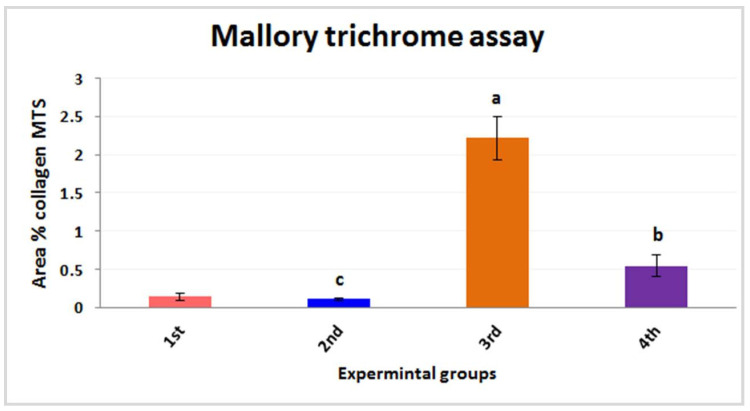
The quantification of fibrous histomorphometric observations for all experimental groups. The experimental groups are as follows; the first group is controlled mice (red column), the second group is PPE-treated mice (blue column), the third group is Fe_2_O_3_-NPs-exposed mice (orange column), and the fourth group is Fe_2_O_3_-NPs plus PPE-treated mice (purple column). Values are expressed as mean ± SE; they differ significantly at *p* < 0.05 by one-way ANOVA followed by Tukey’s multiple comparison post hoc test. The different letters (a, b, c) indicate different levels of significance (*p* < 0.05), “a” indicates high significance (*p* < 0.001), “b” indicates significance (*p* < 0.05), and “c” indicates non-significance (*p* > 0.05.)

Mallory trichrome reaction (MTS) was used for detecting connective tissues, collagenous fibers, reticles, and amyloids that were presented in the hepatic tissues (Figure 5 and Figure 6). It was established that the connective tissues, collagenous fibers, reticles, and amyloids that were distributed in the liver tissues of the first group appeared normally because normal amounts were detected via Mallory trichrome reaction and were positioned mainly in the ground of liver tissues (Figure 5A) (0.14 ± 0.05). In the mouse liver of the second group, the distributions of connective tissues, collagenous, and reticle fibers (Figure 5B) appeared as normal and similar to the first group (0.10 ± 0.02); meanwhile, the hepatic tissues of the third group that were detected using Mallory trichrome reaction revealed a marked increase in collagen fibers. In addition, amyloidosis appeared in the hepatocytes when compared to first group (Figure 5C) (2.22 ± 0.28). In the case of the fourth group, the hepatocytes of the hepatic tissues revealed a mild improvement of the collagen fibers and amyloids using the Mallory trichrome stain (Figure 5D) (0.54 ± 0.14).

#### 3.2.4. TUNEL Assay for Apoptosis

**Figure 7:**
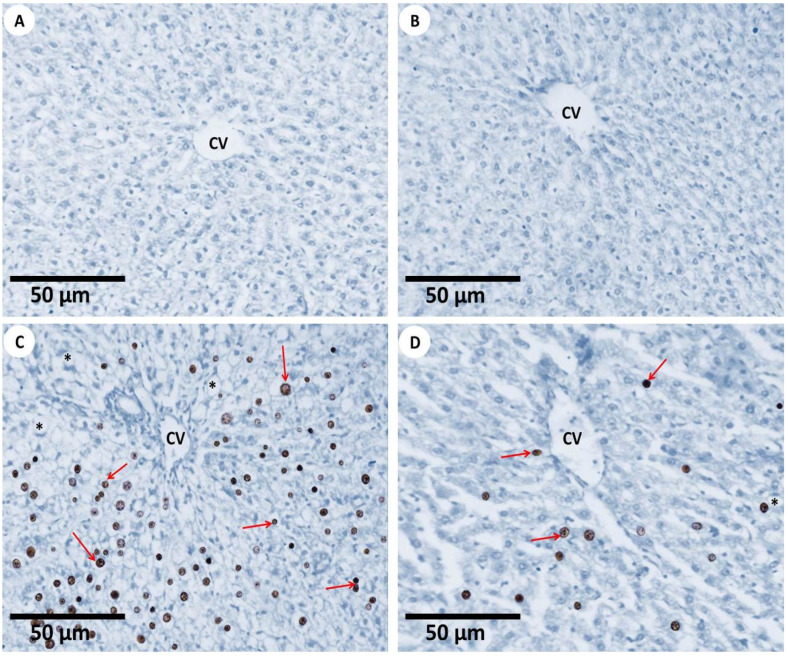
Light explanation microscopy for histochemistry apoptotic cells (**A**) Mouse liver section from the first group displaying no TUNEL-positive apoptotic cells in the healthy tissues of hepatocytes around the central hepatic vein (CV) (TUNEL, 400×). (**B**) Mouse liver section from the second group showing the normal distribution of normal hepatocytes in the hepatic cord found around the central vein (CV) (TUNEL, 400×). (**C**) The mouse liver section from the third group that detected the distribution of apoptotic cells (red arrows) was displayed in the liver tissues after the mice were exposed to Fe_2_O_3_-nanoparticles; the inflammatory cells were found around the central vein (CV), and vacuolation (asterisks) was observed (TUNEL, 400×). (**D**) Mouse liver section from the fourth group showing a cure in the apoptotic cells (red arrows) within the hepatocytes which were located around the hepatic central veins (CV) and a mild appearance of vacuolation (asterisk) (TUNEL, 400×); this shows the usefulness of using PPE in the improvement of toxicity, which was induced in the morphology of the liver tissues of mice exposed to Fe_2_O_3_-NPs.

**Figure 8:**
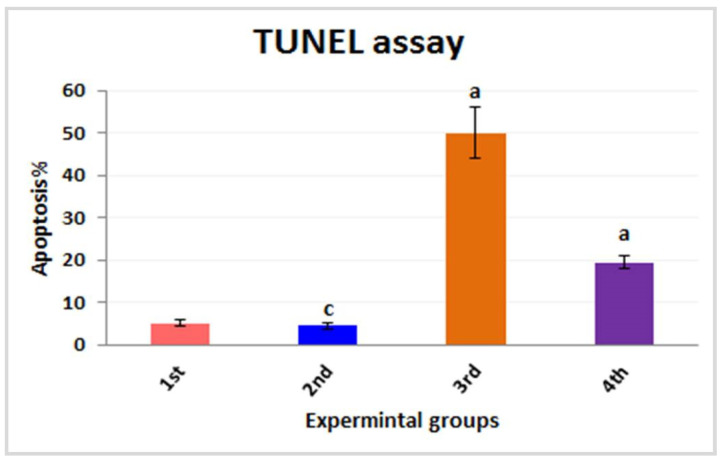
The quantification of fibrous histomorphometric observations for all experimental groups. The experimental groups are as follows; the first group is controlled mice (red column), the second group is PPE-treated mice (blue column), the third group is Fe_2_O_3_-NPs-exposed mice (orange column), and the fourth group is Fe_2_O_3_-NPs plus PPE-treated mice (purple column). Values are expressed as the mean ± SE; they differ significantly at *p* < 0.05 by one-way ANOVA followed by Tukey’s multiple comparison post hoc test. The different letters (a, c) are different levels of significance (*p* < 0.05). The “a” letter indicates high significance, and the “c” letter indicates non-significance.

The findings of the TUNEL assay for the evaluation of apoptosis in all the experimental groups are shown in Figure 7 and Figure 8. The hepatic tissue samples of the first group revealed few apoptotic cells through the TUNEL-positive mechanism (Figure 7A). The liver samples of the second group were similar to those of the first group and also appeared normal (Figure 7B), while the hepatic tissues from the third group detected the apoptotic hepatocytes with a percentage of 49.93%, so the hepatocytes of the third group appeared as the most TUNEL-positive cells (Figure 7C). However, in the case of the fourth group, the hepatic tissues revealed a mild improvement in apoptosis, as revealed by the moderate TUNEL-positive hepatocytes (Figure 7D).

### 3.3. Estimation of Chromosomal Abnormalities

**Figure 9:**
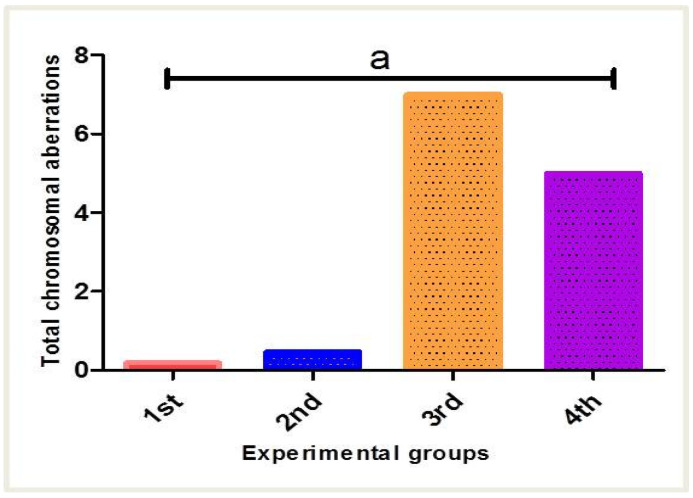
Total chromosomal abnormalities for different experimental groups. The first group is controlled mice (red column), the second group is PPE-treated mice (blue column), the third group is Fe_2_O_3_-NPs-exposed mice (orange column), and the fourth group is Fe_2_O_3_-NPs plus PPE-treated mice (purple column). The level of significance (*p* < 0.05). The “a” letter indicates high significance.

**Figure 10:**
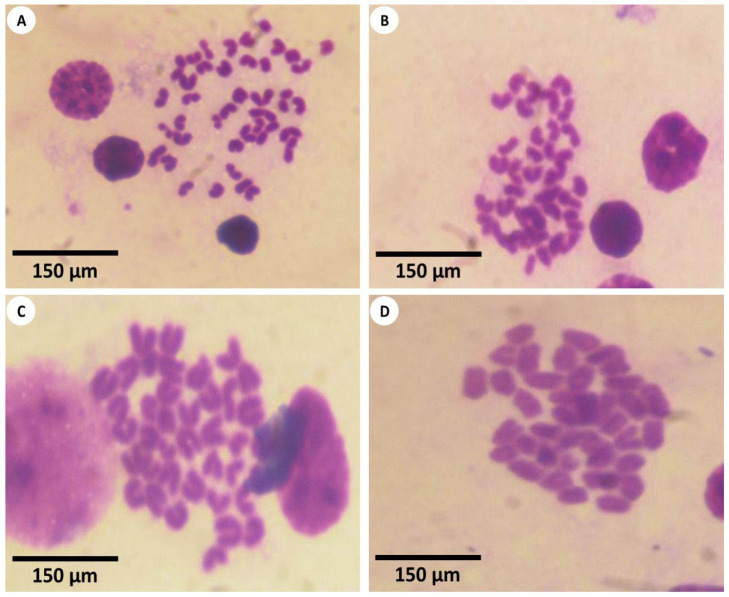
Metaphase smears of different bone marrow cells of mice’s femurs and humeral bones (Giemsa, 1500×) for control mice (first group) and PPE-treated mice (second group) that appeared in the following: (**A**,**B**) revealed control mice chromosomes of the first group with forty detectable normal chromosomes. In addition, (**C**,**D**) revealed normal chromosomes from mice of the second group (PPE) that are similar to the chromosomes of the first group.

**Figure 11:**
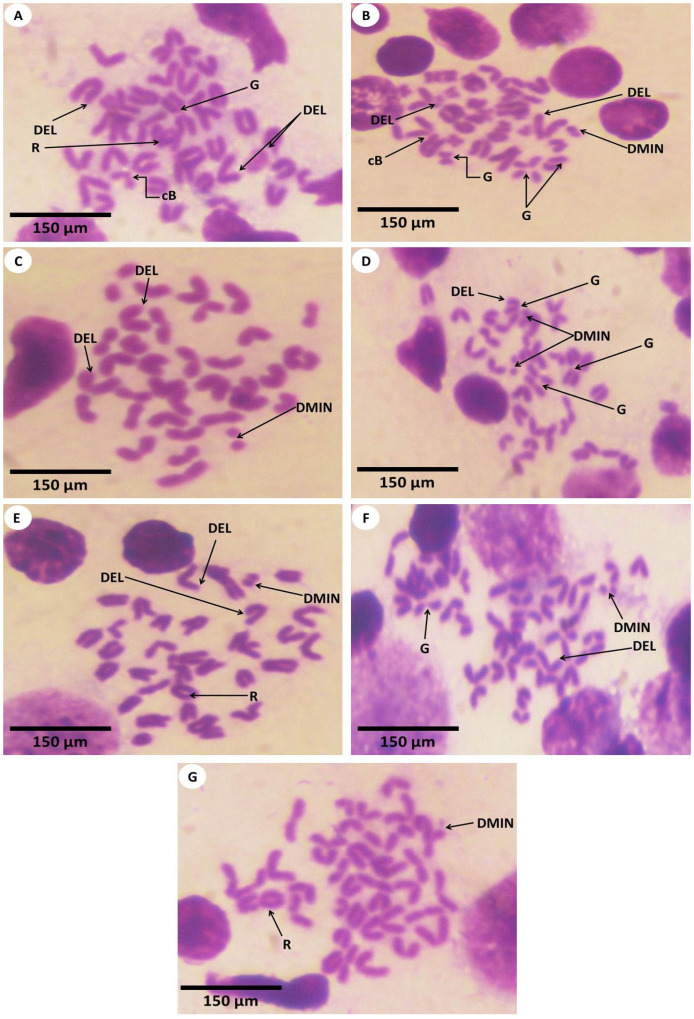
Metaphase smear of different bone marrow cells of mice’s femurs and humeral bones (Giemsa, 1500×) that were presented in the following figures: (**A**–**D**) exhibited abnormalities of chromosomes from mice that were exposed to Fe_2_O_3_-NPs (third group) and that displayed the deletion of chromosomes (DEL), a chromatid gap (G), double minute chromosomes (DMIN), a break of chromatid (cB), and ring chromosomes (R). In addition, the (**E**–**G**) figures from Fe_2_O_3_-NPs plus PPE-treated mice (fourth group) showed ring chromosomes (R), the deletion of chromosome (DEL), double minute chromosomes (DMIN), and a chromatid gap (G).

In the current study, the total chromosomal aberrations of all the experimental groups were statistically analyzed and are shown in Table 3 and Figure 9, Figure 10 and Figure 11. In this context, investigations of chromosomes are a very critical point in determining the genotoxicity of Fe_2_O_3_-NPs and could be used as a diagnostic tool in the observation of toxicity. It was depicted that the experimental mice of both the first and second groups revealed forty intact normal chromosomes without any numerical or structural aberrations (Figure 10A–D). Meanwhile, the chromosomes of the third group displayed a significant increase in the number of chromosomal aberrations including gaps, breaks, deletions, chromosomal rings, and double-minute chromosomes (Figure 11A–D). On the other hand, treatment with PPE in combination with Fe_2_O_3_-NPs was represented by the fourth group and alleviated the toxicity of Fe_2_O_3_-NPs, which was reflected by the improvement in the structure of chromosomes and the appearance of a few chromosomal abnormalities (Figure 11E–G). The statistical analysis of the total types of chromosomal abnormalities from different experimental groups is shown in Table 3. The chromosomal abnormalities were indicated statistically by significant mean ranks and Chi-Square values according to the Kruskal–Wallis H test; the means of these chromosomal abnormalities in all the experimental groups were evaluated compared to the first group. It was declared that the total number of chromosomal aberrations showed a highly significant increase in the third intoxicated group, followed by the fourth group, which was administered orally with both Fe_2_O_3_-NPs and PPE; hence, these findings imply the protective effects of PPE against the genotoxicity induced by Fe_2_O_3_-NPs in these experimental mice, as noted in Figure 11.

### 3.4. Effect of PPE and/or Fe_2_O_3_-NPs on mRNA Expression Patterns of TIMP-1, TNF-α, and BAX Genes

**Figure 12:**
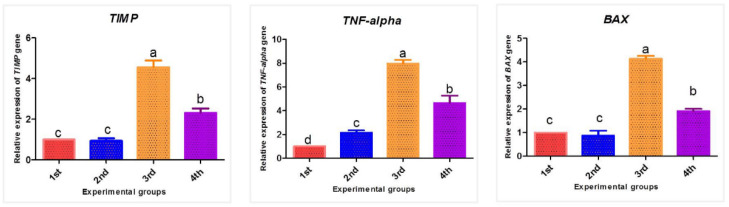
Changes in TIMP-1, TNF-α, and BAX mRNA expression patterns resulting from Fe_2_O_3_-NPs intoxication (30 mg/kg b. wt for 15 days, orally) and PPE (100 mg/kg b. wt) in male albino mice liver tissues. The first group is controlled mice (red column), the second group is PPE-treated mice (blue column), the third group is Fe_2_O_3_-NPs-exposed mice (orange column), and the fourth group is Fe_2_O_3_-NPs plus PPE-treated mice (purple column). Values are expressed as mean ± SE; means differ significantly at *p* < 0.05 by one-way ANOVA followed by Tukey’s multiple comparison post hoc test. The different letters (a, b, c, d) are different levels of significance (*p* < 0.05). The “a” letter indicates high significance, the “b” letter indicates significance, and the “c, d” letters indicate non-significance.

The influence of PPE and/or Fe_2_O_3_-NPs on the TIMP-1, TNF-α, and BAX mRNA expression patterns in the liver tissues of male mice is exhibited in Figure 12. The effect of Fe_2_O_3_-NPs intoxication on the relative mRNA expression was obvious. Studying the expression of some target genes could be used as a proxy marker in evaluating the hepatotoxicity of Fe_2_O_3_-NPs. The mean mRNA expression of the TIMP-1 gene was significantly upregulated in the third group (Fe_2_O_3_-NPs) by 4.56-fold (*p* < 0.05) when compared to the first group (control), suggesting that Fe_2_O_3_-NPs possessed a hepatotoxic effect. Furthermore, the mRNA expression showed significant downregulation in the fourth group (PPE plus Fe_2_O_3_-NPs) by 2.31-fold (*p* < 0.05), followed by the second group (PPE), which is close to first group (control), with a non-significant variation between them (*p* > 0.05). This thus revealed the modulatory effects of PPE on the toxicity of Fe_2_O_3_-NPs. On the other hand, the toxic effect of Fe_2_O_3_-NPs was detected when observing the expression of the TNF-α gene, as its expression served as an indicator of inflammation. Additionally, there was significant upregulation in the third group (Fe_2_O_3_-NPs), with a seven-fold increase in comparison to the first group (control) (*p* < 0.05), followed by the fourth group (PPE plus Fe_2_O_3_-NPs) (4.5-fold, *p* < 0.05). Furthermore, the PPE possessed an antidotal effect against the toxicity of Fe_2_O_3_-NPs that is described when observing the expression of the TNF-α gene of the second group (PPE), as it showed a significant downregulation by two-fold (*p* < 0.05), followed by the first group. In addition, the apoptotic effects of Fe_2_O_3_-NPs upsurged the mRNA expression of the BAX gene in the third group (Fe_2_O_3_-NPs) by 4.00-fold compared to the first group (*p* < 0.05); meanwhile, BAX gene expression was modified in the fourth group (PPE plus Fe_2_O_3_-NPs) to be 1.91-fold with a significant decrease (*p* < 0.05), followed by the second group (PPE), which is similar in expression to the first group (control), with non-significant variation between them (*p* > 0.05). This thus revealed the protective effect of PPE against hepatotoxicity. The stability of the GAPDH gene was assessed during the thermal cyclic conditions of all the experimental samples.

### 3.5. Iron Ion Metal Parameter

**Figure 13:**
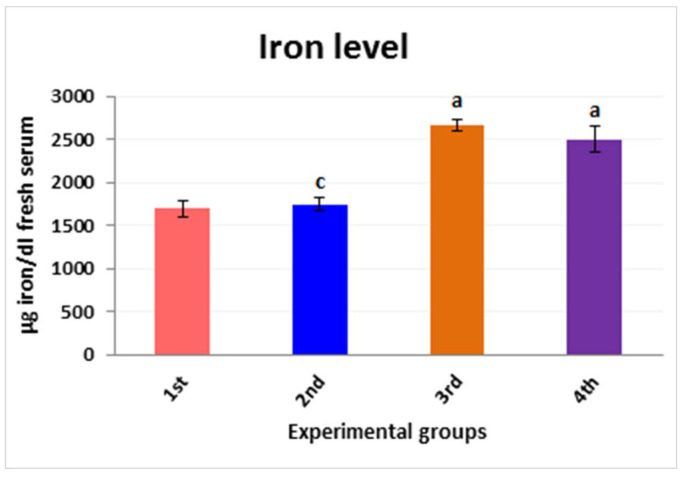
Iron level for mice in the fresh serum of the different experimental groups: the first group is controlled mice (red column), the second group is PPE-treated mice (blue column), the third group is Fe_2_O_3_-NPs-exposed mice (orange column), and the fourth group is Fe_2_O_3_-NPs plus PPE-treated mice (purple column). Values are expressed as mean ± SE; they differ significantly at *p* < 0.05 by one-way ANOVA followed by Tukey’s multiple comparison post hoc test. The different letters (a, c) are different levels of significance (*p* < 0.05). The “a” letter indicates high significance, and the “c” letter indicates non-significance.

The iron level for mice in the fresh serum of the different experimental groups was determined. Our results confirmed the aggregation of iron ions in serum; these levels were significantly increased in the third group in comparison with the first group (2662 ± 70.46 µg/dL to 1693.2 ± 98.40 µg/dL), This parameter was modified regularly using PPE (100 mL/kg) plus Fe_2_O_3_-NPs (fourth group). The level of Fe was noticed to be decreased to 2497.6 ± 153.11 µg/dL. These results show the improved activity of the effect of pomegranate peel extract (PPE) on the liver toxicity caused by iron oxide nanoparticles in mice, as illustrated in Figure 13.

### 3.6. Biochemical Oxidative Stress Parameters

**Figure 14:**
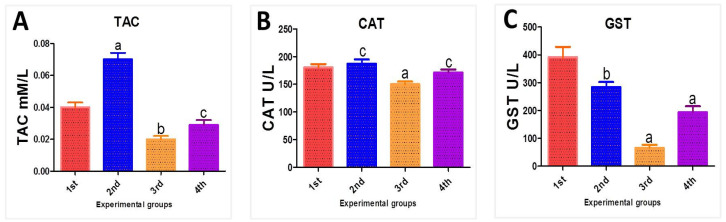
(**A**) Total antioxidant capacity level “TAC” in fresh mice serum, (**B**) Catalase level (CAT) in mice fresh serum, and (**C**) Glutathione S-transferase level (GST) for mice serum of the different experimental groups: the first group is controlled mice (red column), the second group is PPE-treated mice (blue column), the third group is Fe_2_O_3_-NPs-exposed mice (orange column), and the fourth group is Fe_2_O_3_-NPs plus PPE-treated mice (purple column). Values are expressed as mean ± SE; they differ significantly at *p* < 0.05 by one-way ANOVA followed by Tukey’s multiple comparison post hoc test. The different letters (a, b, c) are different levels of significance (*p* < 0.05). The “a” letter indicates high significance, the “b” letter indicates significance, and the “c” letter indicates non-significance.

The biochemical markers are used to make a full description of the hepatotoxicity that was induced by Fe_2_O_3_-NPs and to spotlight the antioxidant properties of PPE. The enzymatic activities of TAC, CAT, and GST were assessed in the serum samples of the experimental mice. The PPE-treated mice (second group) reported levels of antioxidant parameters that were similar to those of the control mice (first group). Meanwhile, the drawbacks of Fe_2_O_3_-NPs intoxication were very clear, as evidenced by the decreased levels of oxidative stress biomarkers. Briefly, there was a significant decrease in the TAC level in the serum samples of mice in the third group compared to the first group—from 0.04 ± 0.003 mM/L serum to 0.02 ± 0.002 mM/L serum. Hence, this agrees with the hypothesis of hepatotoxicity. Our results were also confirmed by detecting the levels of CAT; these levels significantly decreased in the third group in comparison with the first group (180.75 ± 5.02 U/L to 150.16 ± 3.88 U/L), and this decrease tended to be restored to normal in the second group, indicating the antioxidant property of PPE. Finally, to further inform the toxicity of Fe_2_O_3_-NPs, the levels of GST were measured, and they were significantly decreased in the third group from 391.99 ± 36.48 U/L to 65.80 ± 10.58 U/L. In other words, the modulatory effect of using PPE to alleviate the toxicity of Fe_2_O_3_-NPs was observed. These parameters were modified regularly by the use of PPE (100 mL/kg) plus Fe_2_O_3_-NPs (fourth group). The levels of TAC were noticed to increase up to 0.029 ± 0.003 mM/L, the CAT antioxidant activities displayed a significant increase to 171.28 ± 3.42 U/L, and the levels of GST were markedly increased to 194.03 ± 21.17 U/L; these results show the enhancing activity of PPE, as illustrated in Figure 14.

## 4. Discussion

This study sought to identify the antioxidant and hepatoprotective properties of PPE against the hepatotoxicity brought on by Fe_2_O_3_-NPs, as well as the changes in antioxidant biomarkers, hepatostructural modifications, chromosomal aberrations, and TIMP-1, TNF-α, and BAX gene expression patterns in response to PPE treatment.

Magnetic resonance imaging (MRI) is one biological application that has seen an upsurge in the utilization of nanoparticles recently [39]. As MRI contrast agents, Fe_2_O_3_-NPs in particular have been employed [40]. Due to Kupffer cells’ stronger uptake of them compared to other cell types, they have been extensively studied for liver imaging [41]. In addition, the routinely supplied Fe_2_O_3_-NPs in the blood are first taken up by the liver, which is aided by the phagocytic system through endocytosis in the liver’s Kupffer cells. Kupffer cell lysosomes break down Fe_2_O_3_-NPs, releasing free iron that alters the iron homeostasis [42]. Proteins such as ferritin and hemosiderin, which are later utilized by the body, are formed from the storage of this free iron in the body. The body may detect an excess of iron, which causes the generation of reactive oxygen species (ROS) when the ability of these proteins to retain iron is surpassed [43]. After 15 min, nanoparticles were found in the early endosome; they were also found in the late endosomes after 30 and 60 min [44]. 

In this work, mice were gavaged with Fe_2_O_3_-NPs. Hepatocytes’ coagulative necrosis, vacuolar degeneration, pyknosis, karyorrhexis, and karyolysis were signs of the nanoparticles’ significant hepatotoxic effect. Additionally, PPE clearly plays a part in modifying this hepatotoxicity, even though it has several benefits. However, a number of studies [45] found that the size and quality of nanoparticles could have negative effects on biological systems. Different nanoparticles could produce free radicals and cause oxidative damage, which altered how cells functioned [27]. Additionally, the liver carries out a number of metabolic tasks for the body, including the metabolism of xenobiotics and hepatotoxic medications [46]. Although their biosafety is a matter of contention, the liver plays a crucial job in removing nanoparticles from the bloodstream [47].

Hepatic sinusoids were dilated by Fe_2_O_3_-NPs, and the endothelium protecting the liver’s major vein was harmed. These results were in line with those of other investigations that had noted hepatocyte destruction, inflammatory cell infiltration, bleeding in the liver tissue, and sinusoidal dilatation [48,49]. In the cytoplasm of hepatic tissues, another investigation discovered several inflated lysosomes and a damaged, rough endoplasmic reticulum [48]. Hepatocyte degeneration, liver tissue hemorrhage, inflammatory cell infiltration, and hepatic sinusoid dilatation were all the results of the hepatic fibrosis brought on by Fe_2_O_3_-NPs. This fibrosis, often referred to as fibrous aggregation, results in irregularities in the hepatocyte texture over time and progresses to cirrhosis [50,51]. Furthermore, Fe_2_O_3_-NPs propagate the mitochondrial protein nitration and mitochondrial DNA damage, and change the mitochondrial permeability [52].

However, PPE can correct the histological flaws brought on by Fe_2_O_3_-NPs. PPEs and their components have been shown to influence cellular proliferation and inflammation [53]. The strands of hepatic tissues, the endothelial lining of the hepatic central vein, the size of the hepatic sinusoids, and the appearance of the Kupffer cells all improved when the experimental mice were fed PPE. Previous research supported PPE’s beneficial effects on the hepatocytes in the hepatic strands [54,55].

In other words, the hepatic tissues increased free radical aggregation which was facilitated by Fe_2_O_3_-NPs, which led to excessive apoptosis, vacuolated degeneration, inflammatory cell infiltration, steatosis, and blood vessel engorgement. Fe_2_O_3_-NPs had an apoptotic effect on hepatic tissues, which was consistent with other research [56,57]. Further, Fe_2_O_3_-NPs aggregated immunological macrophages in the hepatic tissues and increased the size and quantity of functional Kupffer cells, which triggered inflammatory immune responses [58]. However, some earlier investigations on Fe_2_O_3_-NPs differed from ours. Fe_2_O_3_-NPs were reported to not result in any harm, inflammation, or apoptosis [59]. However, the naturally occurring compounds included in PPE that may change the pro- and anti-apoptotic proteins may negate the stimulated apoptosis [60]. Additionally, PPE reduced oxidative stress and scavenged free radicals to control the hepatic fibrosis brought on by several hepatotoxic substances [55,61].

Fe_2_O_3_-NPs’ genotoxicity was observed by observing how DNA damage and chromosomal abnormalities were affected [52]. Fe_2_O_3_-NPs were found to have genotoxic effects on human blood culture cells, including sister chromatid exchange and chromosomal abnormalities [62,63,64,65,66]. It was discovered that Fe_2_O_3_-NP poisoning enhanced the levels of DNA damage in the skin epithelium and the epithelial cells of the lung in an in vitro model of toxicity [62]. Fe_2_O_3_-NPs can intercalate between DNA base pairs and bind to DNA, resulting in DNA damage [63]. Our results are consistent with a prior study that found that treatment with iron salts significantly increased the rate of chromatid and chromosomal aberrations, as well as DNA damage, in rat bone marrow cells [51]. Fe_2_O_3_-NPs caused chromosomal abnormalities in rats, including ring chromosomes, chromatid breakages, and dicentric and acentric chromosomes [64], which were consistent with our findings. Meanwhile, it was evident from the reduction in the levels of chromosomal abnormalities in the group that received Fe_2_O_3_-NPs + PPE that PPE therapy had a mitigating impact against this genotoxicity (fourth group).

Chromosome abnormalities and the results of histopathological profiles could provide a complete picture of the hepatotoxicity of Fe_2_O_3_-NPs. It was demonstrated that the intoxicated group with Fe_2_O_3_-NPs (third group) had significantly higher levels of TIMP-1 expression, demonstrating the strong hepatotoxic characteristics of these NPs. The communication that takes place between activated hepatic myofibroblasts and liver macrophages results in the production of tissue inhibitors of metalloproteinases 1 (TIMP-1) [65]. Therefore, because TIMP-1 expression is mostly correlated with the level of hepatic fibrosis, it was enhanced in liver tissues and serum during hepatic fibrogenesis in liver-diseased individuals and experimental animal models of hepatic fibrogenesis [66]. The most significant controllers of hepatic fibrosis are, hence, matrix metalloproteinases (MMPs) and TIMPs [67]. TIMP-1 is overexpressed in liver tissues during hepatic fibrogenesis, which is consistent with our findings, and its expression is directly related to the stage of hepatic fibrosis [68]. In the meantime, the usage of Fe_2_O_3_-NPs considerably elevated TNF-α expression, highlighting the NPs’ effects on the liver. The findings in this context were supported by earlier research that clarified the role of TNF-α in the initiation and development of hepatic injury. Hepatocyte apoptosis, which controls the onset of hepatotoxicity in lipopolysaccharide and liver damage, has been found to be significantly influenced by TNF-α [69]. Additionally, the upregulation of TNF-α in the group of people who had consumed Fe_2_O_3_-NPs suggested that it had a significant role in liver fibrosis and inflammation by activating NF-KB, which is an inflammatory trigger [70]. To put it another way, TNF-α has been demonstrated to have protective effects during liver regeneration, but its role during liver injury is different. At this moment, we learned that TNF-α’s concentration controls how it works. Furthermore, the increased levels of TNF-α exacerbate the lipo-polysaccharides that cause liver injury. Additionally, it stimulated liver cell death at high concentrations, although TNF-α may encourage liver cell survival at low quantities [15].

It was found that the expression of the BAX gene increased in response to Fe_2_O_3_-NPs intoxication, while this upsurge significantly decreased as a consequence of using PPE, this extract contains polyphenols and antioxidant compounds. The apoptosis process is defined as a physiological and biological process that is necessary for regular progress and the preservation of homeostasis conditions. Meanwhile, the important properties of apoptosis include cell wrinkling, membrane damage, chromatin compaction, and DNA destruction [71,72]. Additionally, noticeable damage to the inherited genetic material of the cell can activate numerous pathways, leading to apoptosis [73]. The regulation of apoptosis process is very complicated and comprises various proteins; also, BAX proteins promote apoptosis. Once the cell is subjected to apoptosis-causing agents such as Fe_2_O_3_-NPs, BAX is conveyed from the cytoplasm to the mitochondrial membrane and performs the alteration in the permeability of the outer membrane [74]. In contrast to our findings, it was verified that Fe_2_O_3_-NPs caused downregulation in the expression of the BAX gene and apoptosis by decreasing the production of free radicals and increasing the survival of cells [75]. Controversially, the initiation of BAX during apoptosis does not always involve an increase in gene expression, and this expression does not change with the advancement of chronic liver damage [54]. The effect of PPE on the expression of hepatic damage indicators such as TIMP-1, TNF-α, and BAX genes was obvious, their downregulation following PPE treatment indicates the potent anti-inflammatory and anti-apoptotic properties of this extract.

In this study, there was a significant downregulation in the contents of TAC, the activities of CAT, and GST enzymes after Fe_2_O_3_-NPs intoxication. Additionally, antioxidant enzymes are an animal’s first line of defense against free radicals [76]; they trigger processes that neutralize free radicals and ROS [77]. Liver enzymes are important markers of damaged liver cells. Their levels are increased during numerous liver diseases [46], although cells have a powerful antioxidant defense system to intercept ROS. However, if the biological systems fail to nullify the overwhelming ROS, they will lead to the oxidation of biomolecules such as proteins, lipids, and DNA [45]. In our study, it was found that Fe_2_O_3_-NPs induced significant hepatotoxic effects through the generation of ROS, as evidenced by a significant reduction in TAC activities, and these ROS might be detoxified by CAT and SOD by accelerating the decomposition of superoxide radicals into H_2_O_2_, which had been degraded into H_2_O and O_2_ by CAT. The hepatotoxic effects of Fe_2_O_3_-NPs resulted from the elevation of free radicals and a reduction in intracellular antioxidant activities. Additionally, this oxidative stress-induced damage elevated lipid peroxidation in the cell membrane and leaked liver enzymes [63,78]. In line with our results, the use of elevated concentrations of Fe_2_O_3_-NPs caused damage to hepatocytes and upsurged the hepatic enzymes [79]. 

It has been postulated that the activities of hepatic TAC and CAT enzymes were dramatically reduced after exposure to Fe_2_O_3_-NPs at a dose of 100 mg/kg b. wt. in rats [80]. The TAC activity in human blood culture was reduced by varying doses of Fe_2_O_3_-NPs poisoning [81]. Additionally, it is a genotoxic and cytotoxic agent since it causes apoptosis and oxidative DNA damage [63,82]. In good agreement with our findings, rats inebriated orally with 150 mg/kg b. wt. had a lower level of CAT in their serum [83]. Hence, PPE boosts CAT activity in a rat model. Therefore, it increased the expression of CAT, SOD, and GPx genes and modified the toxicity that was induced in the hepatic tissues of rats. These findings could be attributed to the antioxidant effects of this extract [84]. Additionally, PPE could modulate the toxicity of hepatic substances and enhance the decreased levels of the antioxidants CAT and GST in intoxicated rats [85]. The antioxidant effects of pomegranate were similar to those of ascorbic acid, vitamin E, and carotene because of their mixing of a broader array of polyphenols, giving a wide range of action against many free radicals [55]. It was reported that acute toxicity and an LD_50_ test could decide the range of doses and determine the therapeutic index of a herbal extract [86]. In addition, the ethanolic extract of pomegranate fruits and seeds had no toxic effects at a single dose orally administered to mice, and no mortalities were detected at the end of a 24 h period, so the LD_50_ was thought to be greater than 2000 mg/kg b. w.t [87]. Meanwhile, the LD_50_ of the intraperitoneally injected *Punica granatum* extracts was reported to be 731.1 mg/kg in mice [88]. The findings of this study agree with previous reports showing that some flavonoids from plants and fruits are potent O_2_^•−^ scavengers [89]. Moreover, several studies have shown that the pharmacological effects of flavonoids have been related to their antioxidant activity through scavenging OH^•^ and O_2_^•−^ and chelating metal ions [90]. Consequently, the supplementation of PPE alleviates the oxidative injury of the hepatic tissues and enhances the texture and function of the liver in rats subjected to hepatotoxicity [90], this occurs due to the four main components of PPE: terpenoids, polyphenols, nitrogen, and sulfur-containing compounds [91].

## 5. Conclusions

Our experimental study confers promising possibilities for the use of natural substances such as pomegranate peel extract (PPE) against the hepatotoxic effects of iron oxide nanoparticles (Fe_2_O_3_-NPs). This work concluded that the administration of Fe_2_O_3_-NPs to mice prompted the occurrence of oxidative damage as well as injuries in the hepatic tissues, as evidenced by the abnormalities in the iron level, antioxidant parameters, histopathological tissues, histochemistry of fibers, and genetic indices, as well as the decrease in some antioxidant parameters, the increased abnormalities of chromosomes, and the upregulation of the expression of TIM-1, TNF-α, and BAX genes. Finally, the use of PPE alleviates the liver damage induced by Fe_2_O_3_-NPs due to its antioxidant, anti-apoptotic, and anti-inflammatory effects.

## Figures and Tables

**Table 1:** Experimental design for different groups.

Experimental Groups	Design/Dose
1st Group	Ten mice received physiological saline solution only.
2nd Group	Ten mice were administered with 100 mg/kg b. wt. of PPE daily by a stomach feeding tube for 4 weeks.
3rd Group	Ten mice were intoxicated by 30 mg/kg b. wt. of Fe_2_O_3_-NPs suspension for 10 doses daily by a stomach feeding tube, followed by 20 days without receiving 30 mg/kg b. wt. of Fe_2_O_3_-NPs
4th Group	Ten mice were given 100 mg/kg b. wt. PPE plus 30 mg/kg b. wt. of Fe_2_O_3_-NPs, as described for the second and third groups.

**Table 2:** Primer sequences used in real-time quantitative PCR reaction.

Target Gene	Primer Sequences	NCBI Gen Bank Accession No.	References
TIMP-1	F: 5-CCCAGAAATCAACGAGA-3 R: 5-TGGGACTTGTGGGCATA-3	NM_011593.2	[35]
TNF-α	F: 5-GACAGTGACCTGGACTGTGG-3 R: 5-TGAGACAGAGGCAACCTGAC-3	NM_001278601.1	[36]
BAX	F: 5-CTACAGGGTTTCATCCAG-3 R: 5-CCAGTTCATCTCCAATTCG-3	XM_011250780.4	[37]
GAPDH	F: 5-GAGAAACCTGCCAAGTATG-3 R:5-GGAGTTGCTGTTGAAGTC-3	XM_036165840.1	[38]

**Table 3:** The abnormal types of chromosomes for different experimental groups.

		Chromosomal Abnormalities				
		Chromatid Gap	Chromatid Break	Deletion	Ring Chromosome	Double Minute Chromosome
Experimental groups	Control	4.60	5.50	3.60	7.10	6.50
	PPE	6.40	5.50	7.40	3.90	4.50
	Fe_2_O_3_-NPs	13.00	16.00	18.00	18.00	18.00
	PPE plus Fe_2_O_3_-NPs	18.00	15.00	13.00	13.00	13.00
	Chi-Square	16.489	15.350	17.498	16.982	17.502
	*p*-value	0.001	0.002	0.001	0.001	0.001

## Data Availability

The data generated or investigated during this study are obtainable from the corresponding author on reasonable request.
